# Hospital discharge on the first compared with the second day after a planned cesarean delivery had equivalent maternal postpartum outcomes: a randomized single-blind controlled clinical trial

**DOI:** 10.1186/s12884-021-03873-8

**Published:** 2021-06-30

**Authors:** Parvin Ghaffari, Raziyeh Vanda, Shahintaj Aramesh, Leila Jamali, Fatemeh Bazarganipour, Mohammad Amin Ghatee

**Affiliations:** 1 Department of Gynecology and Obstetrics, Yasuj University of Medical Sciences, Yasuj, Iran; 2 Social Determinants of Health Research Center, Yasuj University of Medical Sciences, Yasuj, Iran; 3 Department of Parasitology, Yasuj University of Medical Sciences, Yasuj, Iran

**Keywords:** Cesarean, Early discharge, Maternal outcomes

## Abstract

**Objective:**

Determining the effect of discharge time after elective cesarean section on maternal outcomes.

**Methods:**

This study is a randomized clinical trial that performed on 294 women who undergo elective cesarean section. The patients were randomized in two groups by simple randomization method: Group A (discharge 24 h after cesarean) and group B (discharge for 48 h after cesarean). In both groups, during the first 24 h, they received intravenous antibiotic (cefazolin as routine order) and pethidine at the time of pain. The patients were discharged with the hematinic and mefenamic acid. The main outcome variables were satisfaction of the patient, surgical site infection, separation of incision, endometritis, urinary tract infection, gastrointestinal complications, rehospitalization, secondary postpartum hemorrhage and pain of the patient on discharge day, one and six weeks after cesarean.

**Results:**

Satisfaction scores and pain score at discharge day, one and six weeks after discharge were not significant different in the study groups (*P* > 0.05). Another key finding of this paper was no significant difference in the incidence of surgical site infection, separation of incision, endometritis, urinary tract infection, gastrointestinal complications, rehospitalization, secondary postpartum hemorrhage at one and six weeks after discharge in the study groups(*P* > 0.05).

**Conclusion:**

The time of discharge can be reduced to 24 h after surgery if the mother to be at good general condition, the vital signs are stable, the patient has no underlying problem and disease, and it is financed for the patient and the health system.

## Introduction

Cesarean delivery (CS) is one of the major surgeries in midwifery and its consequences and complications are a major concern of health services. The rate of cesarean delivery worldwide has increased from 21% in 1997 to 33% in 2008 [1]. In the United States, cesarean delivery occurs in one-third of deliveries and women are hospitalized 3–4 days after the procedure. According to The American College of midwifery and gynecology shorter discharges are a choice if the baby is ready to go home, though, the mother should have basic requirements such as normal blood pressure, no symptoms of infection, and adequate pain control [2]. Postpartum stay at hospital is steadily declining in the UK and other countries due to cost savings. Rising hospital costs are one of the factors in early discharge [3].

Several studies have evaluated early discharge after cesarean delivery. Some of study proposed that the length of hospital stay is probably longer after CS (average 3–4 days) than vaginal delivery (average 1–2 days). But according to National Institute for Health and Care Excellence (NICE). (2019), women who are recovering well, are apyrexialand do not have complications, should be discharged early (after 24 h) and followed at home because this is not related to the readmission of the baby or mother. However, other studies have shown that short stays may not leave enough time to diagnose, or treat complications, which in turn can increase morbidity and mortality [4–6]. Fasuba et al. (2000) evaluated the reduction in hospital stay after cesarean delivery and concluded that early discharge may decrease some of the psychological and economic concerns associated with surgery, which is highly acceptable [7]. In another study, Umbeli et.al (2012). evaluated patient”s satisfaction and mortality associated with elective cesarean delivery with 24 h postpartum, and the researchers reported that short stays after Cesarean delivery was associated with greater patient”s satisfaction and no increase in maternal mortality compared to the control group [8]. Early discharge will be reducing hospital care and patient”s costs and improves patient satisfaction. Concerns about early discharge may also include increased discharge, early termination of breastfeeding and increased parental anxiety. Therefore, based on previous studies shows that postpartum stay in hospital, especially in Iran, is not yet documented, limited study available, the present study is an attempt to fill this research gap to describe discharge time after elective cesarean section on the maternal postpartum outcomes.

## Material and methods

### Design and data collection

This study is a randomized clinical trial that performed on 294 women who undergo elective cesarean section in the Gynecology ward of Imam Sajjad Hospital in Yasuj, Iran during 2018–2019.

According to Tan et al. (2012) [9],(P1:0.52; p0: 0.36; α = 0.05; β = 0.80) sample size was estimated at 147 people per group:

$$ n=\frac{{\left[{Z}_{1-a/2}\sqrt{2\overline{P}}\left(1-\overline{P}\right)+{Z}_{1-\beta}\sqrt{P_0\left(1-{P}_0\right)+{P}_1\left(1-{P}_1\right)}\right]}^2}{{\left({P}_1-{P}_0\right)}^2} $$

### Sample characteristics

The inclusion criteria were desire to participate in the study, age less than 35y, elective CS in previous pregnancy, singleton pregnancy, gestation 37 weeks or greater, maternal BMI below 30 (according to weight before pregnancy). The exclusion criteria were having any maternal co-morbidities (i.e. such as immune deficiency, diabetes, hypertension, heart disease, pulmonary and blood disorders), having severe intra-operative or immediate postoperative complications (such as the need for blood transfusion for any reason, fetal anomaly, intolerance to oral liquid diet, postoperative fever), taking any prescription medication, lack of follow-up.

### Description of intervention

Patients were divided into two groups using simple random sampling: Group A) hospital discharge after CS cesarean section for 24 h) and group B) hospital discharge after CS cesarean section for 48 h).

In both groups, during the first 24 h, cefazolin was administered intravenously (according to the hospital routine) and pethidine was administered during pain. Patients with hematinic and mefenamic acid were discharged from the hospital. It should be noted that no additional procedure, i.e. closing the fallopian tubes was performed.

The primary outcome was patient satisfaction with hospital discharge and pain intensity. Secondary outcomes included surgical site infection, incision separation, endometritis, urinary tract infection, gastrointestinal complications, hospital readmission, delayed postpartum hemorrhage at one and six weeks after hospital discharge.

Follow-up examinations included detailed histories and screening systems to examine patients’ wounds for evidence of incision site, drainage from incision, fever symptoms fever symptoms, separation of incision, after additional visits to any medical facility for incision problems or potential related concerns. Participants’ vital signs were measured and urine analysis was performed to assess for urinary tract infections.

This study registered at https://en.irct.ir/user/trial/39789/view with IRCT registration number IRCT20160524028038N3 in 30/05/2019.

### Measures

- A check-list was used to collect demographic and delivery information (age, BMI, smoking status, previous C/S, gravidity, gestational age, C/s duration). BMI was measured by weigh (kg)/Height^2^ (m).
- Outcomes including patient satisfaction with their discharge time,surgical site infection, separation of incision, endometritis, urinary tract infection, gastrointestinal complications, rehospitalization, delayed postpartum hemorrhage, severity of pain in one and six weeks after discharge of hospital on discharge day, one and six weeks after cesarean were assessed as followed:

1. Pain and satisfaction: we used Wong-Baker faces scale to measure pain and satisfaction. This scale is used in people aged three years old and more. The scale is graded with even numbers (0,2,4,6,8 and 10) including six smiles where the zero smiley indicates no pain, the second smiley indicates ‘hurts a little bit’, fourth means ‘hurts a little more’, sixth represents even more problem, eighth indicates ‘hurts a whole lot’, and the tenth shows ‘hurts worst’ [10, 11]. Its validity and reliability are approved [12]. Pain and satisfaction were measured at discharge day, one and six weeks after discharge of hospital.
2. Surgical site infection: having following criteria as Infection within 30 days after operation, partial or total wound dehiscence, presence of purulent or serous wound discharge with induration, warmth/erythema, tenderness.
3. Separation of incision: any defect in the skin incision of at least 1 cm.
4. Endometritis: Temperature ≥ 38 on 2 separate occasions; Clinical diagnosis (≥one clinical observation) including abnormal uterine tenderness on bimanual examination in absence of other clinical or laboratory findings suggestive of another source of infection, concomitant foulsmelling discharge, tachycardia, leukocytosis.
5. Urinary complications including distension of bladder and urinary tract infection (as > 10^5^ bacteria per mL urine).
6. Gastrointestinal complications: as Nausea, constipation, Abdominal distention and pain
7. Postpartum hemorrhage (PPH): The generally accepted definition of secondary PPH is significant blood loss that occurs between 24 h and 12 weeks postpartum [13]. Unlike primary PPH, the quantity of blood loss is not specifically described in literature [14, 15]. The severity is often defined by the need for surgical intervention or blood transfusions [16]. Therefore, in our research, we used the need for surgical intervention or blood transfusions criteria for assess the severity of PPH. Moreover, we used Wong-Baker faces scale for severity of hemorrhage.

### Statistical analysis

Demographic data of the groups were expressed as mean ± SD or number (percent) and comparison of these data was performed by t-test. The normality of the distributions was evaluated using the Kolmogrov-Smirnov test. The statistical program for Social Sciences (SPSS, version 21; SPSS, Chicago, IL). *P* values were set as 0.05 for all analyses. There were no missing values. Therefore, no missing imputation technique was used. This manuscript was prepared in accordance with STROBE guidelines for observational studies.

## Results

### a) Baseline characterizes of participant

**Fig. 1:**
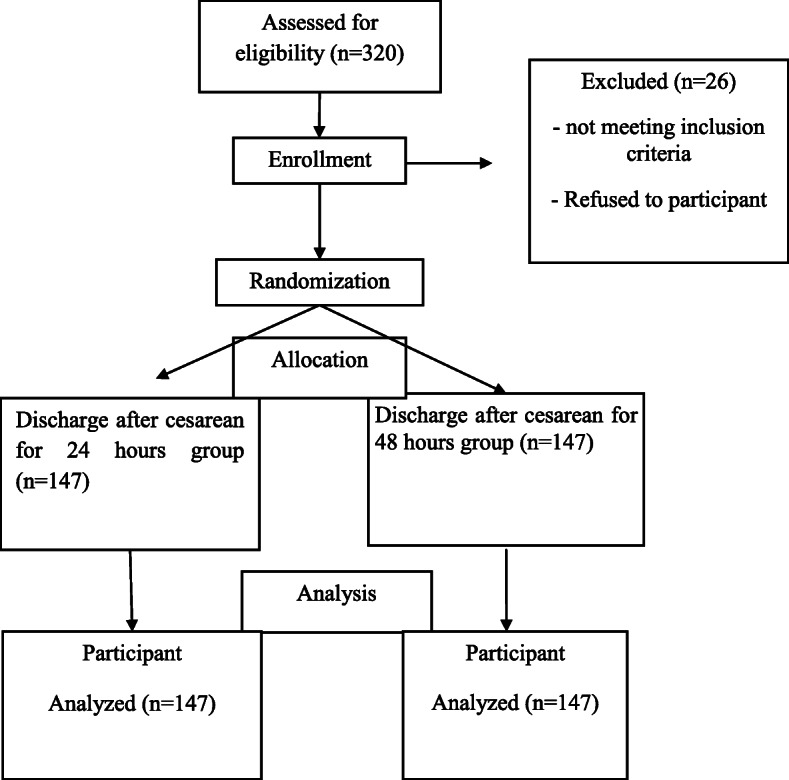
The process of allocating participants during 2018–2019

We assessed 320 subjects for eligibility, among them 26 were excluded for not meeting the inclusion criteria. 294 recruited patients randomly divided to two groups (147 subjects in each group) Group A (discharge 24 h after CS) and group B (discharge 48 h after CS). The process of allocating participants during 2019-02-19 until 2019-07-21 is shown in Fig. 1.

Socio-demographic and clinical characteristic of the patients are presented in Table 1. There was no significant difference between study groups (*P* > 0.05).

**Table 1:** Socio-demographic and clinical characteristic of the patients

Variable	A *n* = 147	B *n* = 147	*P* value*
Age (year) *	29.49 ± 4.07	29.46 ± 3.82	0.94
BMI*	25.41 ± 12.77	23.49 ± 2.99	0.07
C/s duration (min) *	65.00 ± 0.08	65.00 ± 0	0.31
Gestational age (day) *	272.20 ± 10.20	274.59 ± 2.63	0.06
Gravity*	1.34 ± 0.31	1.27 ± 0.69	0.36
smoking status**	5 (3.40)	3 (2.04)	0.12
previous C/S**	68 (46.25)	60 (40.81)	0.97

*mean (SD),T test; ** N(%),X2

### b) Maternal postpartum outcomes between groups

There was no significant difference in the incidence of surgical site infection, separation of incision, endometritis, urinary tract infection, gastrointestinal complications, rehospitalization, secondary postpartum hemorrhage at one and six weeks after discharge in the study groups(*P* > 0.05). We did not observed any surgical intervention or blood transfusions for controlling secondary postpartum hemorrhage between groups.

The satisfaction scores and pain score at discharge day, one and six weeks after discharge were not significant different in the groups (*P* > 0.05). (Table 2).

**Table 2:** Maternal postpartum outcomes in discharge day, one and six weeks after discharge in the study groups

Variable			A *n* = 147		B *n* = 147		*P* value*
			yes	no	yes	no	
Surgical site infection	1 week after discharge		1 (0.7)	146 (99.3)	ـــ	147 (100)	*p* = 0.31
	6 weeks after discharge		2 (1.4)	145 (98.6)	3 (2)	114 (98)	*p* = 0.65
Endometritis	1 week after discharge		0	147 (100)	0	146 (99.3)	0
	6 weeks after discharge		0	147 (100)	0	147 (100)	–
Separation of incision	1 week after discharge		1 (0.7)	146 (99.3)	0 (0)	147 (100)	0.3
	6 weeks after discharge		2 (1.4)	145 (98.6)	1 (0.7)	146 (99.3)	0.56
Rehospitalization	1 week after discharge		4	143	2	145	0.06
	6 weeks after discharge		3	144	3	144	–
Urinary tract infection	1 week after discharge		14 (9.5)	133 (90.5)	17 (11.6)	130 (88.4)	*p* = 0.56
	6 weeks after discharge		3 (2.04)	144 (97.95)	2 (1.36)	145 (98.63)	*P* = 0.43
Gastrointestinal complications	Abdominal pain	1 week after discharge	6 (4.1)	141 (95.9)	5 (3.4)	142 (96.6)	*p* = 0.75
		6 weeks after discharge	3 (2.04)	144 (97.99)	1 (0.69)	146 (99.31)	P=/53
	Abdominal distention	1 week after discharge	19 (12.9)	128 (87.1)	14 (9.5)	133 (90.5)	*p* = 0.35
		6 weeks after discharge	2 (1.36)	145 (98.63)	2 (1.36)	145 (98.63)	–
	Constipitation	1 week after discharge	9 (6.1)	138 (93.9)	14 (9.5)	133 (90.5)	*p* = 0.27
		6 weeks after discharge	3 (2.04)	144 (97.99)	2 (1.36)	145 (98.63)	P=/55
	Nausea	1 week after discharge	ـــ	147 (100)	ـــ	147 (100)	ـــ
		6 weeks after discharge	0 (0)	147 (100)	0 (0)	147 (100)	–
Secondary postpartum hemorrhage severity	1 week after discharge		6.34 ± 1.21		6.49 ± 1.87		0.41
	6 weeks after discharge		0		0		–
Satisfaction score	Day of discharge		3.30 ± 1.23		3.82 ± 3.45		0.08
	1 week after discharge		2.29 ± 1.50		2.54 ± 1.68		0.17
	6 weeks after discharge		1.85 ± 1.23		1.86 ± 1.18		0/92
Pain score	Day of discharge		1.43 ± 0.11		1.23 ± 0.10		0.17
	1 week after discharge		1.85 ± 1.68		1.99 ± 1.69		0.47
	6 weeks after discharge		1.73 ± 0.70		1.36 ± 0.76		0/98

## Discussion

In uncomplicated cesarean delivery, the average hospital stay is two to four days, but studies have shown that earlier discharge may be appropriate in women and infants who are correctly selected [17]. Complications of this surgery include infection, wound and fascia opening, metritis, abscess, gastrointestinal complications such as cramping, bloating, constipation, and urinary tract infections [17].

The aim of this study was to evaluate the effects of 24-h and 48-h post-cesarean section discharge. According to the present study, the time of discharge can be reduced to 24 h after surgery if the mother to be in good general condition, the vital signs are stable and the patient have no underlying problem and disease. Against, the study evidence shows four common health problems, including fatigue, insomnia, breast problems and constipation among Turkish women after early discharge [18]. Women who are discharged early after giving birth are significantly more likely to be depressed than those who stay longer in the hospital [19, 20]. Therefore, previous research suggests that early postpartum discharge has a negative effect on women’s health. In contrast, several studies show that early postpartum discharge of healthy mothers and full-term infants does not appear to have any side effects if discharge is provided with a policy of offering accompanied by at least one referral to a nurse-midwife at home [19]. Our findings have been shown there was no significant difference in the incidence of surgical site infection, separation of incision, endometritis, urinary tract infection, gastrointestinal complications, rehospitalization, secondary postpartum hemorrhage at one and six weeks after discharge in the study groups. In agreements with this study. Katusiime et al. reported that early discharge on the second day after delivery of uncomplicated cesarean section was acceptable for infants and healthy mothers without the need for a home visit [21]. Brown et al. who reported that early discharge for mothers and children does not have a detrimental effect on breastfeeding or postpartum depression [22]. In a study in South Africa in 2017, they examined the effect of 48 h and 33 to 57 h postpartum discharge and reported no significant difference in complications between the two groups [23]. In Tan et al. (2012)., day 1 discharge compared with day 2 discharge after a planned cesarean delivery resulted in equivalent outcomes (exclusive breastfeeding, unscheduled maternal or infant medical consultations, rehospitalizations, maternal antibiotic use, and maternal well-being, anxiety, and depression status) [9].

Since the 1990s, in most health care centers, measuring patient satisfaction has been considered as a way of receiving patients’ views and opinions about their care [24, 25]. In this study, patient satisfaction was assessed in both groups after the intervention. Another key finding of this paper is that satisfaction and pain score was not significantly different at discharge day, one and six weeks after discharge of hospital. In the study which conducted by that Tan et al. (2012)., the patient satisfaction evaluated in one day postoperative and two days postoperative groups and they reported there was no significant difference between the two groups [9].

Our study has several strengths including randomization (and successful randomization since both groups similar), greater sample size using standard measures for accurate assessment of pain and satisfaction and the inclusion control group for comparison. There are some limitations. Our study was limited to an only low-risk population from hospital this, in turn, limits generalization the finding to the whole population and external validity. Further studies with larger sample size recruited from a less homogenous population are recommended.

## Conclusion

In conclusion, according to the present study and data from previous studies, if the mother is in good general condition, with stable vital signs without any underlying problem and disease, the time of discharge from the hospital can reduced to 24 h after surgery.

## Data Availability

The primary data for this study is available from the authors on direct request.
